# *SLCO4A1* expression is associated with activated inflammatory pathways in high-grade serous ovarian cancer

**DOI:** 10.3389/fphar.2022.946348

**Published:** 2022-08-29

**Authors:** Stephanie Koller, Jonatan Kendler, Jasmine Karacs, Andrea Wolf, Caroline Kreuzinger, Isabel Von Der Decken, Felicitas Mungenast, Diana Mechtcheriakova, Wolfgang Schreiner, Andreas Gleiss, Walter Jäger, Dan Cacsire Castillo-Tong, Theresia Thalhammer

**Affiliations:** ^1^ Department of Obstetrics and Gynecology, Translational Gynecology Group, Comprehensive Cancer Center, Medical University of Vienna, Vienna, Austria; ^2^ Department of Pathophysiology and Allergy Research, Center for Pathophysiology, Infectiology and Immunology, Medical University of Vienna, Vienna, Austria; ^3^ Center for Medical Statistics, Informatics, and Intelligent Systems, Medical University of Vienna, Vienna, Austria; ^4^ Department of Pharmaceutical Sciences, Division of Pharmaceutical Chemistry, University of Vienna, Vienna, Austria

**Keywords:** high-grade serous ovarian cancer, *SLCO4A1*, OATP4A1, NF-kB, ABCC3, *SLCO4A1-AS1*, inflammatory signaling pathways

## Abstract

Patients with high-grade serous ovarian cancer (HGSOC) have a very poor overall survival. Current therapeutic approaches do not bring benefit to all patients. Although genetic alterations and molecular mechanisms are well characterized, the molecular pathological conditions are poorly investigated. Solute carrier organic anion transporter family member 4A1 (*SLCO4A1*) encodes OATP4A1, which is an uptake membrane transporter of metabolic products. Its expression may influence various signaling pathways associated with the molecular pathophysiological conditions of HGSOC and consequently tumor progression. RNA sequencing of 33 patient-derived HGSOC cell lines showed that *SLCO4A1* expression was diverse by individual tumors, which was further confirmed by RT-qPCR, Western blotting and immunohistochemistry. Gene Set Enrichment Analysis revealed that higher *SLCO4A1* level was associated with inflammation-associated pathways including NOD-like receptor, adipocytokine, TALL1, CD40, NF-κB, and TNF-receptor 2 signaling cascades, while low *SLCO4A1* expression was associated with the mitochondrial electron transport chain pathway. The overall gene expression pattern in all cell lines was specific to each patient and remained largely unchanged during tumor progression. In addition, genes encoding ABCC3 along with SLCO4A1-antisense RNA 1, were associated with higher expression of the *SLCO4A1*, indicating their possible involvement in inflammation-associated pathways that are downstream to the prostaglandin E2/cAMP axis. Taken together, increased *SLCO4A1*/OATP4A1 expression is associated with the upregulation of specific inflammatory pathways, while the decreased level is associated with mitochondrial dysfunction. These molecular pathophysiological conditions are tumor specific and should be taken into consideration by the development of therapies against HGSOC.

## Introduction

Although progress in the treatment of high-grade serous ovarian cancer (HGSOC) has been made, the poor survival rate (<40%) of patients has not changed substantially over the last few decades (Bowtell et al., 2015). The majority of patients is usually diagnosed at advanced stages (FIGO stage III or IV) in which the tumor has disseminated beyond the ovaries and pelvic organs to the peritoneum and abdominal organs (Lisio et al., 2019). Peritoneal metastases are also associated with the formation of ascites, which increases the intraperitoneal pressure and blocks circulation (Tan et al., 2006).

At the molecular level, HGSOC is characterized by mutations in *TP53*, which contributes to the genetic instability of cancer cells and drug resistance (Ahmed et al., 2010; Zhou et al., 2019). Patients are operated and treated with platinum-base drugs. After initial successful therapy, the early onset of drug resistance contributes to the poor prognosis of patients. Defining cancer relevant cellular pathways in HGSOC is essential to develop efficient new therapies (Cornelison et al., 2017).

To meet the increasing demand of nutrients, oxygen and the removal of metabolic products from proliferating tumor cells, specialized transport proteins are required (Pizzagalli et al., 2021). They also mediate the uptake of endogenous regulators such as tissue factors and hormones. Previous studies revealed that members of the organic anion transporting polypeptide (OATP) family, which are encoded by the *SLCO* genes, are expressed in various secretory functional tissues and at biological barriers, including the small intestine, liver, lung, and kidney. This is congruent with their proposed function in the absorption, distribution and excretion of metabolic products, drugs and xenobiotics. Upregulation of different members of this family in a variety of tumors indicate a role for *SLCOs*/OATPs in cancer (Obaidat et al., 2012; Buxhofer-Ausch et al., 2013; Nakanishi and Tamai, 2014; Thakkar et al., 2015).

Among the *SLCO* family, *SLCO4A1* is of particular interest, as it is highly expressed in ovarian cancer (Svoboda et al., 2018). Its expression has been previously associated with inflammation in different tissues (Wojtal et al., 2009; Chen et al., 2014) and in some cancers, e.g., colon cancer (Ban et al., 2017; Buxhofer-Ausch et al., 2019). *SLCO4A1* encoded OATP4A1 acts as a transporter for a number of endogenous OATP substrates, such as steroid hormone conjugates (estrone sulfate), prostaglandins including the major endogenous pro-inflammatory prostaglandin PGE_2_, thyroid hormones and xenobiotics and drugs (Hagenbuch and Gui, 2008). Uptake of pro-inflammatory prostaglandins into cells would result in their enhanced degradation by cytosolic enzymes. Their increased uptake would decrease the concentration of extracellular prostaglandins available for binding and activation of the membrane-bound G-protein coupled receptors (Schuster, 2002). This is important for the development of ovarian cancer. In the normal ovary, the maturation of the follicle and the release of the oocyte are accompanied by the recruitment of activated immune cells and the subsequent secretion of chemokines, cytokines and growth factors for ovarian tissue repair (Duffy et al., 2019). The resulting pro-inflammatory environment is considered to favor the malignant transformation of cells in the ovary and peritoneal cavity (Savant et al., 2018), by which *SLOC4A1*/OATP4A1 might play a role through the regulation of prostaglandins. On the other hand, cytokines and tissue factors produced in the inflammatory environment may alter the expression levels of *SLOC4A1*. Indeed, increased *SLCO4A1* levels were shown in the colon of patients with inflammatory bowel disease (Wojtal et al., 2009). However, in the placenta, bacterial infection did not influence expression of *SLCO4A1* (Petrovic et al., 2015).

To elucidate *SLCO4A1* expression and to establish a possible association between the transporter und cellular signalling pathways in HGSOC, we used 33 patient-derived ovarian cancer cell lines which were demonstrated to reflect the pattern of genes expressed in tumors from individual patients (Kreuzinger et al., 2019).

## Materials and methods

### Materials from patients

**FIGURE 1:**
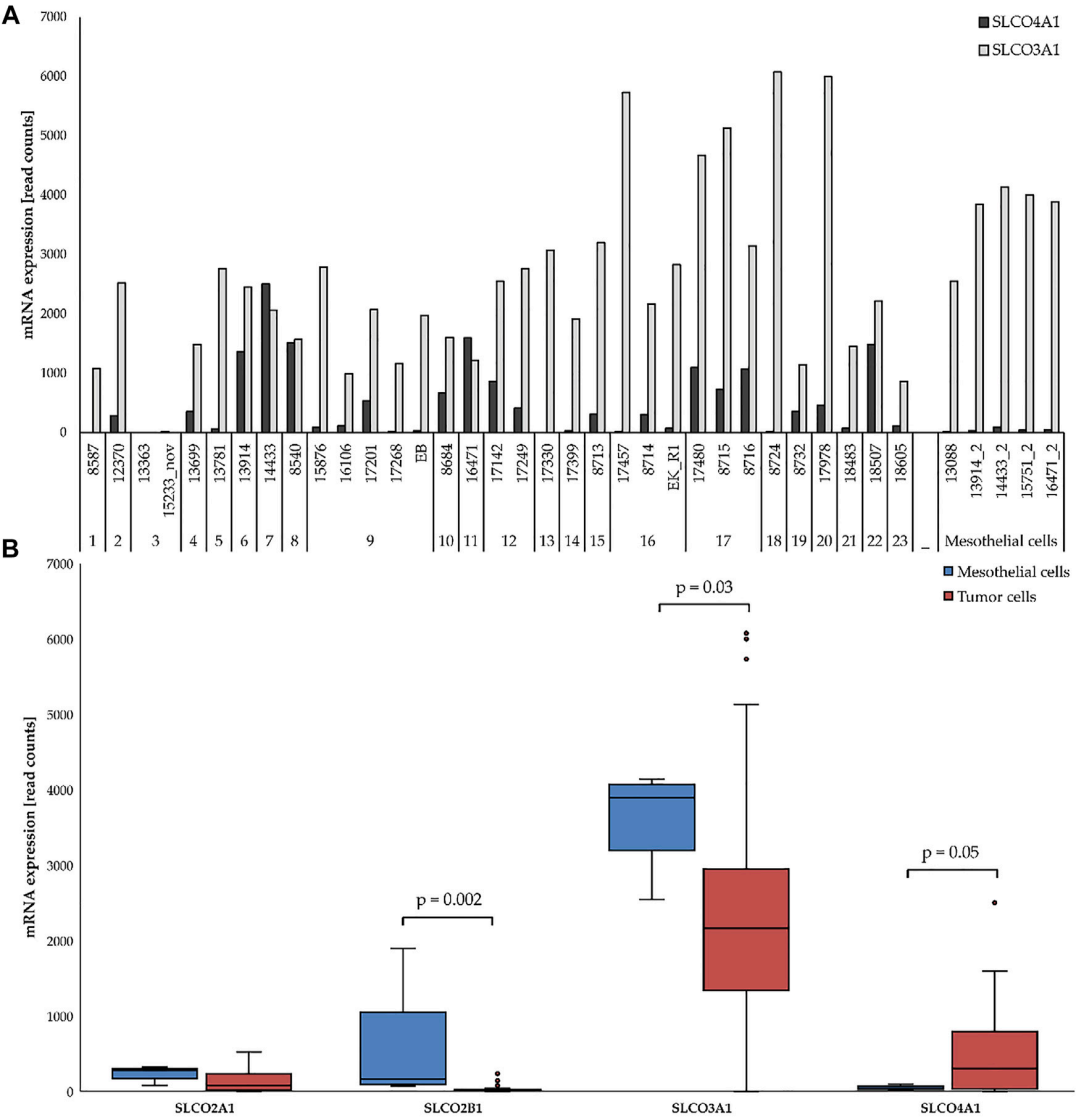
mRNA expression of selected SLCOs in ovarian cancer cell lines (red) and mesothelial cells (blue). **(A)** SLCO4A1 and SLCO3A1 levels in 5 mesothelial and 33 ovarian cancer cell lines from HGSOC patients. Cell lines and the code for each patient are indicated at the bottom. **(B)** Expression of SLCO4A1 and three other genes in the ovarian cancer (red) and mesothelial (blue) group. Boxes include all data from the first to third quartile, with the indicated median. The upper and lower lines indicate the maximum and minimum values excluding any outliers. Outliers are indicated as dots. Statistical significance (*p* < 0.05).

Thirty three patient-derived HGSOC cell lines (from patients 1–23) and 5 primary cell cultures with predominant mesothelial cells (derived from patients 6, 7, 9, 11 and from an additional patient for #13088) (Figure 1A) were previously established and molecularly characterized (Kreuzinger et al., 2019). All information pertaining to these cell lines is available from the Expasy Cellosaurus cell line database (https://web.expasy.org/cellosaurus). RNA sequencing data were previously published (Kreuzinger et al., 2019).

### Cell culture

Cells were cultivated at 37°C and 5% CO_2_ in DMEM Medium supplemented with 10% Fetal Bovine Serum, 10,000 Units/ml Penicillin and 10,000 µg/ml Streptomycin (all from Gibco Life Technologies, CA, United States). Before splitting, cells were rinsed first with PBS and thereafter, trypsinized with 0.05% Trypsin-EDTA (1x) (Gibco Life Technologies) (splitting ratio 1:2–1:4). Cells were stored in liquid nitrogen in medium supplemented with 10% DMSO (Sigma-Aldrich, St. Louis, United States).

### DNA and RNA preparation

AllPrep DNA/RNA Mini Kit (Qiagen, Hilden, Germany) was used for DNA and RNA extractions according to the manufacturer’s instructions. The yield and purity were calculated by measuring the absorbance at 260/280 nm with Nanodrop 1,000 (Thermo Scientific, MA, United States). DNA was stored at 4°C and RNA at −80°C. For cDNA synthesis, the Omniscript Reverse Transcription Kit (Qiagen, Hilden, Germany) was used and the cDNA was stored at −80°C.

### Reverse transcription quantitative PCR

Gene expression of *SLCO4A1* (ENSG00000101187) obtained *via* RNA sequencing was validated by RT-qPCR using the ABI prism 7900HT system (Applied Biosystems; Thermo Fisher Scientific, Inc.) and TaqMan Gene Expression assay for *SLCO4A1* (Hs00249583_m1) (Thermo Fisher Scientific, Inc.). All reactions were performed in duplicate. A cell line with a higher expression of the target gene was used to generate standard curves. By comparing the CT value of a certain sample relative to the standard curve, a relative gene expression value for each sample was calculated and expressed as fold-change. Statistical significance (*p* < 0.05) between the expression of each individual *SLCO* in HGSOC and mesothelial cell cultures was calculated using the Mann-Whitney test (SPSS 27; IBM Corp.). *p* < 0.05 was considered to be statistically.

### Sanger sequencing

To determine the potential influence of gene mutations on gene expression, Sanger sequencing was performed on a particular region that may contain the g.323G>A (p.Val78Ile; Accession No. AB031051.1) mutation. Primer pairs (sense, 5′-AGA​CGT​GAG​CTT​GCT​AAC​CAG-3′; antisense, 5′-TGG​TAG​CTG​TGC​AGG​TCA​TAG-3′) were designed and PCR was performed using DNA samples under the following thermocycling conditions: 40 cycles of 30 s at 94°C, 60 s at 65°C and 80 s at 72°C. PCR products were purified using the GFX PCR DNA kit (Amersham Biosciences Corp.) and sequenced by Eurofins Genomics LLC. The CLC mainwb5 program was used for data evaluation.

### Quantification of protein in cells by western blotting

For protein extraction, the cell pellet was resuspended in cold RIPA buffer (Sigma-Aldrich; Merck KGaA) with proteinase inhibitor (Sigma-Aldrich; Merck KGaA) and incubated on ice for 30 min. The lysates were centrifuged at 12,000 rpm for 20 min at 4°C, after which the supernatant was collected and stored at −80°C. For protein quantification, a Bradford Assay was used. A total of 20 µg protein was mixed with 5X Laemmli Sample Buffer containing 5% β-mercaptoethanol, and heated for 5 min at 95°C. Subsequently, the proteins were loaded on a Mini Protean stain-free Tris-Glycine extended gel (TGX, 8%–16%; Bio-Rad Laboratories, Inc.) and separated at 100 V for 60 min under denaturing conditions. Samples were then transferred onto nitrocellulose membranes at 25 V and 4°C using electrophoresis Power Supply 301 equipment (Amersham Pharmacia Biotech, Inc.). After 24 h, the membranes were blocked with 5% dried milk in TBS-0.1% Tween for 1 h at room temperature. The membranes were then incubated with the following primary antibodies for 60 min at room temperature: OATP4A1 (rabbit IgG; Clone H-145; 1:100; Santa Cruz Biotechnology, Inc.) and GAPDH (Rabbit IgG; Clone 14C10; Cell Signaling Technology, Inc.). After washing the membrane three times with TBS-Tween, the membrane was incubated with anti-rabbit horseradish peroxidase (HRP)-conjugated secondary antibody (Cell Signaling Technology, Inc.) at room temperature for 1 h. Target proteins were detected using the LuminataTM Crescendo/Classico Western HRP Substrate (Merck KGaA) for chemiluminescent detection with FluorChem Q (Alpha Innotech). The blots were evaluated according to their intensity and volume using open-source software Image Lab 6.0.

### Immunohistochemistry

Formalin-fixed paraffin-embedded (FFPE) tumor tissues were obtained from the Department of Pathology, Medical University of Vienna. Informed consents were obtained from all patients prior to sample collection. All processes were approved by the local ethical committee (EK366/2003, EK260/2003). Immunohistochemistry was performed on 3 µm sections cut using the automatic microtome HM355S (Microm International GmbH, Walldorf, Germany). The sections were dried overnight and deposited at −20°C before being further processed. Thereafter FFPE sections were treated with xylol (Fisher Scientific, Loughborough, United Kingdom) and a series of descending ethanol concentrations. To permeabilize the tumor tissue, sections were incubated in 0.5% Triton X (Merck Millipore, Darmstadt, Germany) diluted in PBS for 15 min at RT. After washing in PBS and in distilled water 3 times for 2 min each, antigen retrieval was performed by boiling the slides in a citrate buffer (pH 6) (Merck Millipore) in a microwave oven at 850 W for 2.5 min, which was followed by 160 W for 10 min. They were slowly cooled down to RT for 1–2 h. The endogenous peroxidase was blocked by incubating the slides for 10 min in 3% H_2_O_2_ (Merck Millipore) diluted in methanol (Thermo Fisher Scientific). Afterwards, the samples were washed twice in distilled water, each time for 2 min. To block unspecific binding, the samples were covered with Dako Ultra V Block (Agilent Technologies, Vienna, Austria) for 7 min and then washed in PBS-Tween twice. The primary antibodies were diluted to the optimal concentration in Dako Antibody-Diluent (Agilent Technologies). OATP-E/SLCO4A1 antibody (Rabbit IgG, Clone H-145; at 1:100; Santa Cruz, United States) and Negative Control rabbit IgG (BioCare Medical, CA, United States) were used. The slides were then incubated with the antibody at 4°C overnight.

Then the slides were rinsed and washed twice with PBS-Tween, each time for 3 min. Dako LSAB2 System—HRP was applied in a 3-step procedure, by adding a biotinylated anti-rabbit and anti-mouse immunoglobulin for 30 min, streptavidin conjugated to horseradish peroxidase for 30 min and a substrate-DAB solution for about 5–20 min. The slides were rinsed with PBS-Tween twice, 3 min between each step. The reaction was observed under the microscope and stopped by washing with distilled water for 3 min.

To stain the nucleus, Hematoxylin Gill III (Merck Millipore, Darmstadt, Germany) was added to the sections for 1–2 s and the slides were immediately rinsed in water to stop the reaction. Slides were covered with Kaiser’s glycerol-gelatine (Merck Millipore) and dried at RT.

### Double-immunofluorescence staining

FFPE sections were treated with xylol and descending ethanol concentrations. For antigen retrieval, a citraconic anhydride buffer was used. PBS/Tween containing 5% goat serum was applied for blocking. First, staining was done with the rabbit anti-human antibody against OATP4A1 (HPA030669, Atlas Antibodies AB), and, thereafter, a mouse-monoclonal antibody against cytokeratin 19 (A53-B/A2, Abcam) as a marker for epithelial ovarian cancer cells (Menz et al., 2021) was applied. An Alexa Fluor 488-labelled anti-rabbit antibody and an anti-mouse Alex-Fluor 568-labelled antibody (Abcam, Cambridge, MA, United States) were added as secondary antibodies. For negative controls, the primary antibodies were replaced by non-immunogenic IgG. To stain the nuclei, the sections were incubated for 10 min with DAPI (0.2 µg/ml in PBS). After rinsing with distilled water, the sections were mounted with Fluoromount G and covered by cover slides.

### Gene set enrichment analysis

For the identification of *SLCO4A1* related pathways, the tumor cell line samples were categorized for high and low *SLCO4A1* levels. Eight cell lines demonstrating the highest and eight cell lines with the lowest *SLCO4A1* expression were selected for analysis. In cases where more than one cell line derived from the same patient appeared in the same group, the mean was calculated. GSEA 4.1.0 (https://www.gsea-msigdb.org/gsea/index.jsp) was used for the analyses. The data sets c2.cp.kegg.v7.1.symbols.gmt for KEGG and c2.cp.biocarta.v7.2.symbols.gmt for BIOCARTA were selected from the Molecular Signatures Database (MsigDB: https://www.gsea-msigdb.org/gsea/msigdb/index.jsp).

### Statistical analysis

All statistical analyses were performed using IBM SPSS version 27, except the heatmap, which was generated using R version 3.5.2. The heatmap function in R 3.5.2 was used to hierarchically cluster log-transformed expression values with respect to cell lines and genes. Pearson’s correlation coefficient was used to quantify the correlation between pairs of variables in case of a linear association. Spearman’s correlation coefficient was used otherwise in case of a monotone association. *p*-values < 0.05 are assumed to indicate statistical significance.

## Results

### Characterization of *SLCO4A1*/OATP4A1 in the cell lines

We first compared the RNA levels of selected *SLCOs* in the 33 ovarian cancer cell lines and the five primary cell cultures containing mainly mesothelial cells (Kreuzinger et al., 2019) by RNA sequencing. *SLCO2A1*, *2B1*, *3A1*, and *4A1* were detected at considerable levels in the cell lines (Figure 1B). While levels of *SLCO2A1*, *SLCO2B1* and that of the most abundantly expressed *SLCO3A1* were higher in mesothelial cells than in ovarian cancer cells (*p* = 0.002 and *p* = 0.03, respectively), *SLCO4A1* mRNA levels were higher in ovarian cancer cells (median of 304 read counts) as compared to mesothelial cells (median of 41 read counts) (Figure 1; *p* = 0.05).

The strong correlation between RNA sequencing and RT-qPCR data indicating the reliability of the sequencing data (Supplementary Figure S1; Pearson correlation coefficient: *R* = 0.86; *p* < 0.001).

Sanger sequencing revealed that the heterozygous mutation g.323G>A (p.Val78Ile), previously described in colon cancer and healthy control patients, was present in 2 out of 7 investigated cell lines. This SNP, which is located in the first intracellular loop of the protein distant to the binding site of drugs, was previously shown to have no effect either on the expression levels nor on the uptake of the OATP surrogate substrate sodium-fluorescein (Buxhofer-Ausch et al., 2020).

Comparison with clinical data of patients showed that *SLCO4A1* mRNA level correlated neither with the origin of the tumor cells (either from tumor tissue, *n* = 8; or ascites; *n* = 23; or pleural fluid, *n* = 2) nor with the time of sample collection (at the primary diagnosis, *n* = 20 or during disease progression and chemotherapy, *n* = 13), (*p* = 0.85 and 0.98, respectively). For example, the cell line with the highest *SLCO4A1* expression (2,502 read counts in cell line 14433) and the cell line with a *SLCO4A1* level below the detection limit (cell line 17330) were both derived from tumors of patients at the time of the first diagnosis prior to platinum-based chemotherapy. Furthermore, cell line 13363 and 15233 were derived from patient 3 before and after chemotherapy, respectively and had similar low *SLCO4A1* expression (5 and 20 read counts, respectively).

**FIGURE 2:**
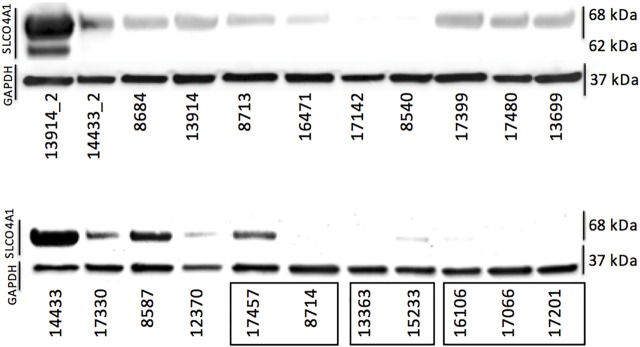
Protein levels of OATP4A1 determined *via* Western blotting. The first two samples (13914_2 and 14433_2) are primary cell cultures containing mostly mesothelial cells. The three clusters on the bottom right (indicated with black rectangles) are cell lines derived from the same patient; all others are single cell lines generated from different patients. GAPDH was used as loading control.

**FIGURE 3:**
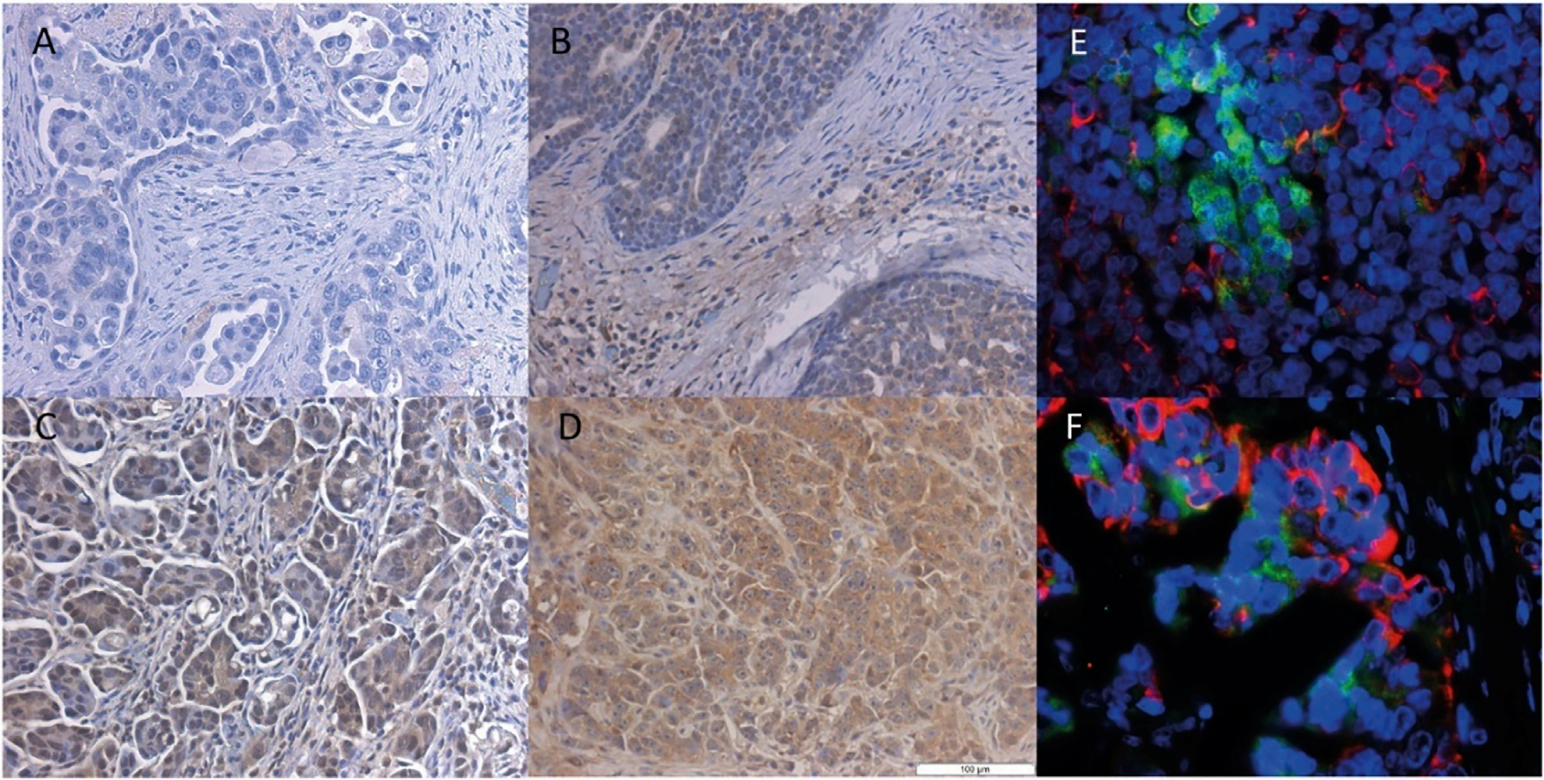
Immunohistochemical staining of the OATP4A1 protein in FFPE samples from HGSOC. Representative IHC staining of ovarian cancer cells with different levels of SLCO4A1/OATP4A1 protein in FFPE samples images **(B–D)**; the negative control FFPE sample is shown in image **(A)**. Immunofluorescence staining of OATP4A1 and CK19 is shown in images **(E–F)** Red fluorescence indicates cytokeratin-19 and green fluorescence OATP4A1; blue indicates DAPI staining of nuclei (magnification ×40).

**FIGURE 4:**
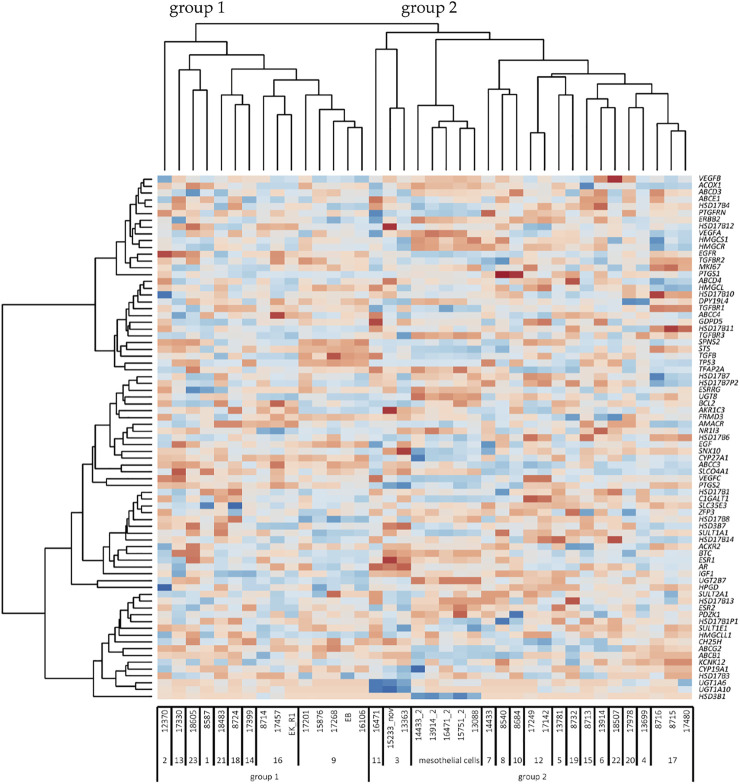
Heatmap of unsupervised clustering analysis (*n* = 38 cell lines). Cell names are indicated below the heatmap. The numbers under the cell line names represent the code used for each patient. Group 1 and 2 indicates the two large cell clusters according to the pattern of genes (dark red, minimum log2 expression; dark blue, maximum log2 expression). On the right-hand side, the 77 genes are indicated. The 5 mesothelial cell lines have low levels of SLCO4A1, but the overall pattern of genes is related to group 2 of ovarian cancer cells. Note that various column clusters are built from cell lines obtained from the same patient.

We also examined the *SLCO4A1* encoded OATP4A1 in the ovarian cancer cell lines by Western blotting and showed OATP4A1 immunoreactive protein in ovarian cancer samples by IHC and double-IF staining. On the Western blot, the immunoreactive band at approximately 70 kDA corresponded to the predicted molecular mass of the transporter (Figure 2). An additional band at 62 kDA in the mesothelial cells 13914_2 may reflect the non-glycosylated protein. The semi-quantitative evaluation of the immunoreactive band shows a moderate correlation between the immunoreactive protein and relative *SLCO4A1* expression levels (Pearson correlation coefficient = 0.52, *p* = 0.024; Supplementary Table S1; Supplementary Figure S1). By IHC staining, we showed that *SLCO4A1*/OATP4A1 in FFPE samples reflects the *SLCO4A1* expression in the tumor cell lines derived from the tumor of the same patient (Figures 3A–D). The transporter was mainly visible on the membrane and in the cytoplasm of the tumor cells (Figures 3B–D), similar as the observation in other tumors including colon carcinoma (Kleberg et al., 2012; Buxhofer-Ausch et al., 2019). As expected, immunoreactive OATP4A1 was not detected in adipose tissues. Double staining with an antibody against CK19 (red color), which was previously shown to be highly present in the ovarian cancer cells (Svoboda et al., 2018; Menz et al., 2021), revealed that OATP4A1 (green color) is rarely detectable in cells together with CK-19 but rather in cells that do not express this marker (Figures 3E,F). This is in line with the findings that cells with high *SLCO4A1* levels have an overall gene expression pattern more similar to that of mesothelial cells (see Figure 4).

### Genes related to *SLCO4A1* levels

To examine the possible relationship between *SLCO4A1* and genes coding for enzymes and transporters and genes in the pathways for prostaglandin and lipid turnover, steroid hormone receptors, and their transcriptional regulators, a set of 77 genes was selected (Supplementary Table S2). Spearman correlation analysis of individual genes indicated that *ABCC3*, an efflux pump from the family of multidrug resistance-related transporters, has a strong and significant positive correlation to *SLCO4A1* in the ovarian cancer cells (*r* = 0.75, *p* < 0.001). Correlation values for all other genes were lower (|*r*|=<0.6).

Unsupervised clustering of the 77 genes revealed two major groups of cell lines (Figure 4). Group 1 with lower levels of *SLCO4A1* consists of 15 ovarian cancer cell lines from nine patients (median *SLCO4A1* levels: 72 read count). Group 2 with significantly higher levels (*p* = 0.005) of *SLCO4A1* (median: 412 read counts) includes the remaining 18 ovarian cancer cell lines from 14 patients and the 5 mesothelial cell lines, the latter with *SLCO4A1* levels of 41 read counts (median). Within the group of mesothelial cells, the gene expression pattern is rather uniform and gives these cells a separate sub-cluster next to three ovarian cancer cell lines from group 2, which are adjacent to group 1 cell lines. The residual ovarian cancer cell lines belonging to group 2 are located at the right side of the diagram. The overall gene expression pattern of group 2 ovarian cancer cells shows a high association with the expression pattern of genes in the mesothelial cells, which are associated with cell metabolism and proliferation.

Importantly, cell lines derived from the same patient had the closest relationship in the gene expression pattern independent of the time of establishment (at the primary diagnosis or later during disease progression and exposure to chemotherapy) as visible from the column clusters. We also show that only minor changes in the overall gene expression pattern during tumor progression and chemotherapy were detectable in the ovarian cancer cell lines, which were derived from the same patients at the time of diagnosis or later after tumor progression and chemotherapy. It seems that some populations of ovarian cancer cells keep their phenotype during tumor progression. Nevertheless, cancer cell populations with other properties and an altered phenotype produced by clonal selection, which takes place in HGSOC (Lambrechts et al., 2016) might get lost during the isolation procedure.

### *SLCO4A1* associated signaling pathways

In order to identify which pathways might be associated with the expression of *SLCO4A1*, eight ovarian cancer cell lines with the highest (median 1,420 read counts) and eight cancer cell lines with the lowest *SLCO4A1* expression levels (median 18 read counts) (*p* = 0.001) were selected to perform the gene set enrichment analysis (GSEA). The database Kyoto Encyclopedia of Genes and Genomes (KEGG) and the BIOCARTA database were used for the analysis.

**FIGURE 5:**
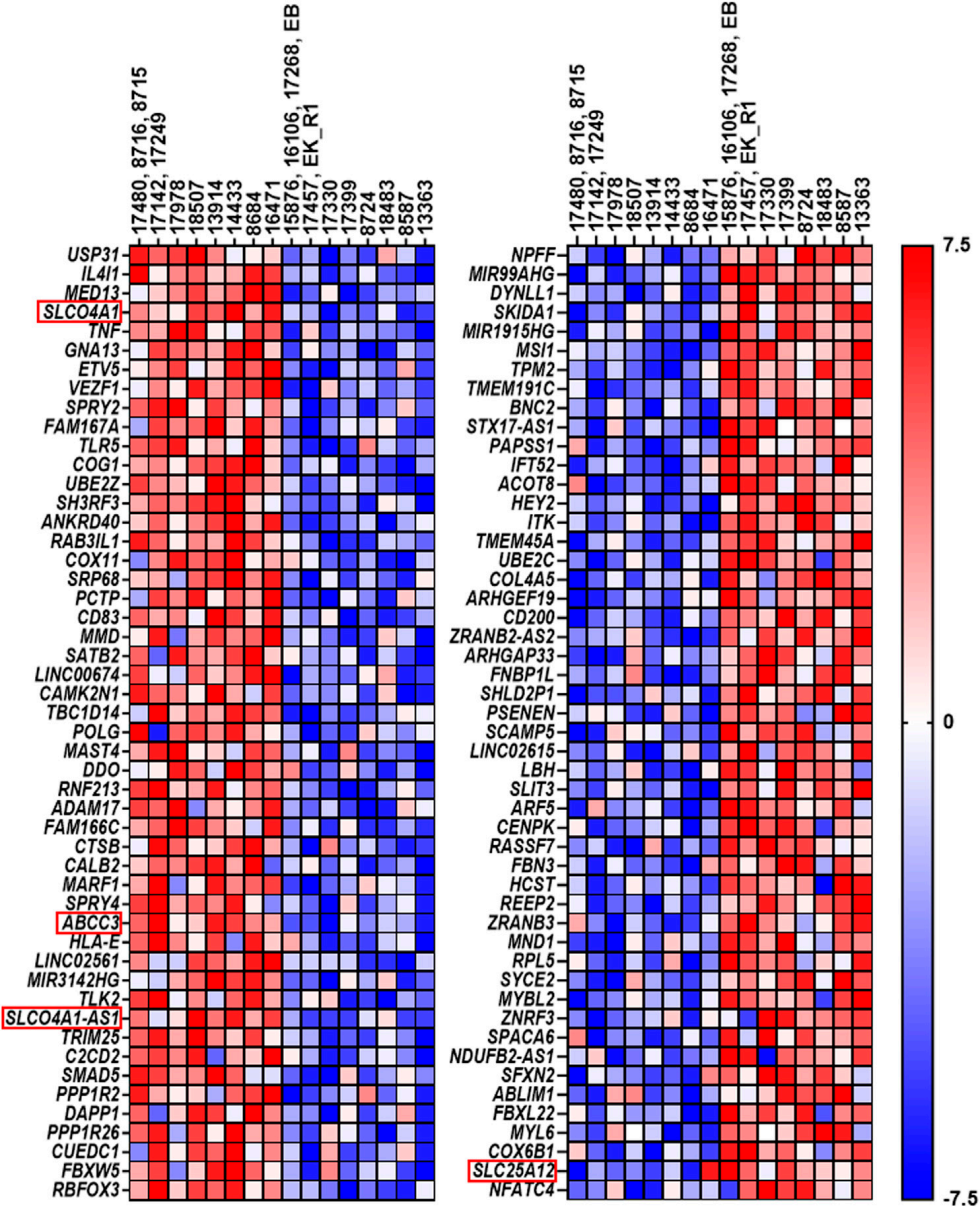
Top 50 genes resulting from gene set enrichment analysis. The left side displays the top 50 genes in cell lines expressing the highest SLCO4A1 level, while the right side shows the top 50 genes, which were upregulated in cell lines expressing the lowest SLCO4A1 level. Each of the two columns is annotated with their respective cell lines. Again, the cell lines on the left side of each column show the higher expressing ones, while the right side shows the cell lines, which express the lowest level of SLCO4A1. In cases where several cell lines were derived from the same patient, the mean value of the RNA expression from these cell lines was calculated and used. Gene expression is normalized for each row; where red indicates higher expression while blue indicates the lower expression of the respective genes (ranked from −7.5 to 7.5).

The resulting heat map is shown in Figure 5. Among the top 50 enriched genes, also *ABCC3* coding for MRP3, an efflux pump for non-conjugated organic anions and glucuronide-conjugates of endogenous and exogenous compounds (Keppler, 2011) was present, suggesting that a close relation between the uptake and efflux by these transporters may exist. Interestingly, also the expression of *SLCO4A1* Antisense RNA 1 (*SLCO4A1-AS1*) was related to high *SLCO4A1* levels.

In the group of ovarian cancer cells with low *SLCO4A1* levels, *SLC25A12* coding for the SLC25A12 transporter was identified among the most significant enriched genes. SLC25A12 is responsible for the calcium-dependent exchange of cytoplasmic glutamate with mitochondrial aspartate (Palmieri et al., 2001) and may therefore contribute to disturbances in the mitochondrial function (Figure 5).

The most significant pathways associated with high and low *SLCO4A1* expression are given in Table 1. The enrichment score (ES) reflects the degree to which the genes are overrepresented at the top or bottom of the entire ranked gene list. The values are normalized to the mean enrichment of random samples of the same size (NES) and range from 2.06 to 1.73. The nominal (NOM) *p*-value estimates the significance of the observed enrichment score for a single gene set and the False-Discovery rate (FDR), which gives the estimated probability that a gene set with a given enrichment score represents a false positive finding (normalized for gene set size). The latter values are normalized for gene set size.

**TABLE 1:** Over-presented pathways in relation with *SLCO4A1* expression.

Database	Pathways	Size	NES	NOM p-val	FDR q-val
Enriched pathways in cell lines with higher *SLCO4A1*					
BIOCARTA	TALL1	13	2.06	0	0.006
	CD40	14	2.02	0	0.011
	NFKB	20	1.97	0	0.015
	TNFR2	16	1.93	0	0.015
	KERATINOCYTE	45	1.94	0	0.018
	HIVNEF	53	1.82	0	0.046
	TID	18	1.83	0	0.047
	RELA	14	1.92	0.002	0.017
	CDMAC	16	1.89	0.002	0.024
	IL1R	30	1.79	0.002	0.052
	41BB	15	1.84	0.004	0.052
	LAIR	15	1.73	0.004	0.068
	DEATH	29	1.76	0.004	0.062
	CSK	16	1.83	0.006	0.071
	PPARA	48	1.83	0.008	0.047
	IL7	16	1.77	0.008	0.059
KEGG	NOD like receptor signaling	54	1.97	0	0.034
	Adipocytokine signaling	60	1.84	0	0.072
	Cell adhesion molecules CAMS	118	1.73	0.002	0.107
	Leishmania infection	64	1.89	0.002	0.069
	Apoptosis	81	1.82	0.002	0.072
	TOLL like receptor signaling	79	1.85	0.002	0.084
	Nature killer cell mediated cytotoxicity	98	1.72	0.006	0.104
Enriched pathways in cell lines with lower *SLCO4A1*					
BIOCARTA	ETC	10	1.93	0	0.032

Size, the numbers of genes included in the pathway; NES, normalized enrichment score; NOM *p*-val, normalized *p*-value; FDR q-val, False discovery rate q-value.

From the 16 pathways identified in the BIOCARTA and the seven in the KEGG analysis associated with higher *SLCO4A1* expression, the TALL-1, CD40, NF-KB and TNFR2 signaling pathways (BIOCHARTA) and the Nucleotide-binding, oligomerization domain (NOD)-like receptors (NLRs) and the adipocytokine signaling pathways (KEGG) were the most prominent ones (FDR < 0.125, *p* < 0.01, Table 1). These particular pathways are related to NF-kB signaling, suggesting a prominent role of the transporter in NF-kB related inflammatory processes.

Lower expression of *SLCO4A1* was significantly associated with the mitochondrial electron transport chain (ETC) pathway, which is important for ATP production and the maintenance of reactive oxygen species (ROS) homoeostasis in cells. This pathway had been identified in many tumors, including in HGSOC and was indicated as an potential therapeutic target (Nayak et al., 2018) (BIOCARTA analysis, Table 1).

## Discussion

The current study aimed at elucidating the expression of *SLCO4A1* in patient-derived ovarian cancer cell lines. These cell lines provide a comprehensive transcriptomic picture in HGSOC and offer the advantage to study gene expression in cells generated during chemotherapy and tumor progression (Kreuzinger et al., 2019).

Different from the four *SLCO* genes (*SLCO3A1*, *2A1*, and *2B1*) with high expression in mesothelial cells, we identified *SLCO4A1* having higher levels in a group of ovarian cancer cell lines compared with mesothelial cells. We confirmed the correlation of the corresponding protein expression by immunohistochemistry and Western blotting.

By comparing levels of *SLCO4A1* with a panel of genes, selected for their role in the regulation of biotransformation, cell proliferation and tumor progression, we identified two groups of cancer cell lines, one with high *SLCO4A1* and the other with low *SLCO41* levels. Surprisingly, for the ovarian cancer cell group with high *SLCO4A1*, the overall pattern for the genes was similar to that of mesothelial cells. The primary mesothelial cell cultures were separated from the ascites, from which the tumor cell lines were generated. Because the mesothelial cells detached from the cultural flasks more quickly than tumor cells under the treatment of Trypsin-EDTA, we enriched both cell populations by collecting cells at different trypsinization time points. These mesothelial cells usually could be passaged for 6–8 times, thus they contain certain percentage of tumor cells. These could be a reason, why they were clustered with tumor cells. In addition, this might reflect dynamic processes during tumor progression, where cancer cells undergo EMT, mesothelial cells might undergo cancer cell–mediated reprogramming for promoting cancer cell metastasis (Davidson et al., 2012). This suggests that altered levels of *SLCO4A1* might be a consequence of cell transformation and/or *SLCO4A1*/OATP4A1 might contribute to the transformation, e.g., by mediating the uptake of specific substrates. Interestingly, studies in colorectal cancer showed that high levels of the LncRNA *SLCO4A1-AS1* gene, which go along with high levels of *SLCO4A1,* promote EMT by activating the Wnt/β-catenin signaling (Wei et al., 2020) and a similar mechanism might take place in HGSOC.

An important finding of the analysis of gene expression in the groups of cells with high or low *SLCO4A1* levels revealed that different signaling pathways important for tissue inflammation and immune response (Micalizzi et al., 2010; Savant et al., 2018) including NF-κB are related to *SLCO4A1* levels. We found also NOD-like receptor, adipocytokine, TALL1 [Tumor Necrosis Factor (Ligand) Superfamily, Member 13b], CD40, and TNFR2 signaling pathways related to high *SLCO4A1*, while low *SLCO4A1* levels show an upregulation of the electron transport chain pathway (ETC) suggesting disturbances in the mitochondrial ATP production, ROS homeostasis and the metabolic pattern (Luo et al., 2020). Various pathways are interconnected *via* the NFκB system controlling inflammatory processes in cancer (Li et al., 2013; Mitchell et al., 2016).

The activation of NOD-like receptors, which belong to the family of pattern-recognition receptors, occurs *via* host-derived damage signals, ATP and ions, as well as pathogen-associated molecules from bacteria and viruses. This activation induces signaling pathways to stimulate the NF-κB -/activator protein 1-dependent expression of various pro-inflammatory cytokines, including IL-1β and IL-18, as well as the interferon regulatory factor-dependent expression of type I interferons (Liu et al., 2019). Tissue factors from the family of adipocytokines that regulate lipid metabolism such as adiponectin and resistin are expressed in ovarian cancer cells and can drive tumor progression (Dogra et al., 2021). A wide range of intracellular signal pathways, including those for apoptosis, cell survival, inflammation and immunity are activated by TNF receptor (TNFR) signaling *via* distinct pathways, including the PI3K-dependent NF-κB pathway, the MAPK cascade and the JNK pathway (Peng et al., 2015). Activated TNF forms a homotrimer by binding to its receptors TNFR1 and TNFR2. After ligand binding, the receptor of TNF facilitates the association of different TNFR associated factor adaptor proteins to initiate the recruitment of signal transducers. Also the other signaling pathways that we identified involve other members of the TNF-superfamily, including TALL1 and CD40, cause a broad variety of responses that influence cytokine secretion and modulate cell growth and survival (Gallagher, 2002).

High *SLCO4A1* levels could be the consequence of an altered cell metabolism in inflammatory tissue. Increased systemic production of pro-inflammatory cytokines due to acute or chronic inflammatory conditions can impact the expression of membrane transporters, although different results are reported. Studies on various *SLCOs*/OATPs revealed that the impact of different cytokines on their expression depends on the tissue and cell type (Svoboda et al., 2011; Hagenbuch and Stieger, 2013). For example, in colon of patients with inflammatory bowel disease, the mRNA expression levels of *SLCO4A1*, and also other *SLCO*s, e.g., *SLCO2B1* were upregulated, while the expression of transporters from other families was downregulated (Wojtal et al., 2009). Downregulation of hepatic SLCOs (*SLCO2B1*, *1B1*, and *1B3*) was observed after interferon-γ application in human hepatocytes (Le Vee et al., 2011).

In our study, low levels of *SLCO4A1* levels were associated with the ETC pathway in which the production of ROS is important, because it influences hypoxia adaptation critical for cell survival and proliferation (Raimondi et al., 2020). Since putative hypoxia-inducible (factor HIF)-1 transcription factor binding sites were identified in the *SLCO4A1* promotor, hypoxia may cause an upregulation of *SLCO4A1,* which was observed in neuroblastoma (Applebaum et al., 2016) and for other *SLCOs* in colon and pancreatic cancer (Han et al., 2013). On the other hand, excessive ROS may lead to irreversible cell damage and activate pathways leading to cell death (Raimondi et al., 2020). This was shown in the human eye, where downregulation of *SLCO4A1* is a marker of photoreceptor cell death during retinal detachment (Delyfer et al., 2011). These results suggested that HGSOC with high or low *SLCO4A1* expression might be associated with disease progression through different mechanisms.

**FIGURE 6:**
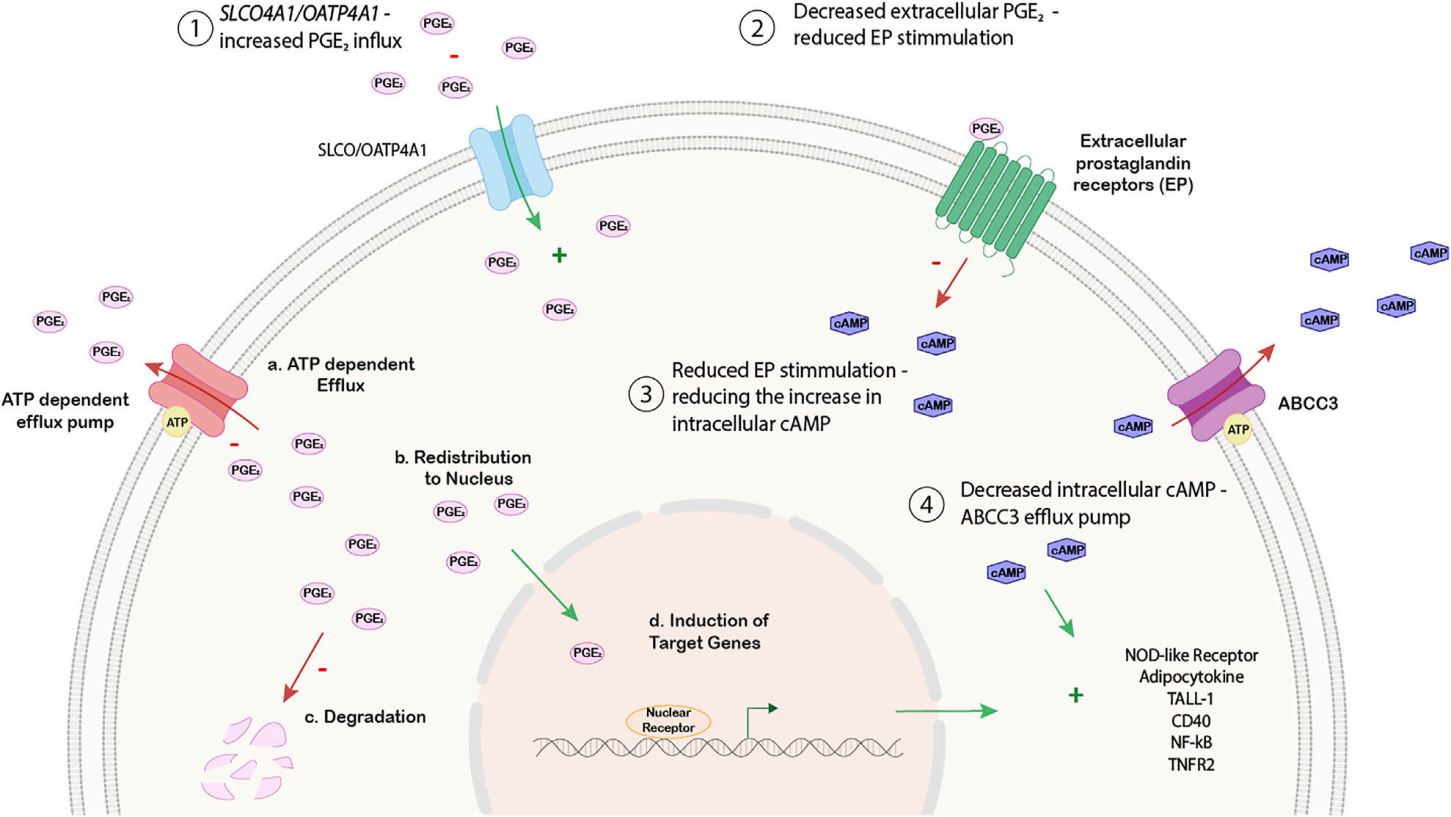
Working hypothesis on the relationship between SLCO4A1 expression and NF-kB associated pathways.

In addition to the regulation of *SLCO4A1* through pathological alterations such as metabolic changes with ROS production and inflammation, *SLCO4A1*/OATP4A1 expression may also change cancer cell metabolism and cell fate by its transport function for endogenous substrates. For example, higher expression of *SLCO4A1* would lead to an increased influx of prostaglandins into cells, causing their increased intracellular degradation and there would be less extracellular prostaglandin available for receptor activation. Subsequently, reduced prostaglandin signaling would lead to reduced receptor activation and signaling *via* cAMP (Menter and DuBois, 2012). Furthermore, upregulation of ABC efflux pumps known to export c-nucleotides (such as ABCC3) would further alter down-stream signaling. A proposed model is shown in Figure 6.

Other substrates for OATP4A1, which could be important for HGSO, are steroid hormone conjugates. Uptake of the estrogen precursor estrone sulfate as the most prominent estrogen in postmenopausal women (Rižner et al., 2017) and its effective conversion to 17ß-estradiol in the cells could lead to an increase in estrogenic signaling and tumor progression in estrogen receptor expressing tumor cells. However, neither pathways for the activation of estrogen sulfate nor estrogen receptor signaling were related to *SLCO4A1* levels in our analysis. Therefore, these pathways are rather unlikely to be related to *SLCO4A1* in the ovarian cancer cell lines.

## Conclusion

In summary, we showed that *SLCO4A1* expression was diverse and specific for individual tumors, which was further confirmed by RT-qPCR, Western blotting and immunohistochemistry. High *SLCO4A1* levels were associated with inflammation-associated pathways including NOD-like receptor, adipocytokine, TALL1, CD40, NF-κB, and TNF-receptor 2 signaling cascades, while lower *SLCO4A1* expression was associated with the mitochondrial electron transport chain pathway. In addition, genes encoding SLCO4A1-antisense RNA 1 and the ABC-efflux pump ABCC3 were associated with higher expression of the *SLCO4A1*, indicating their possible involvement in inflammation-associated pathways that are downstream to the prostaglandin E2/cAMP axis. These molecular pathophysiological conditions are tumor specific and should be taken into consideration when developing therapies against HGSOC.

## Data Availability

The original contributions presented in the study are included in the article/Supplementary Material, further inquiries can be directed to the corresponding author.
