# Supplementary material for: SLCO4A1 expression is associated with activated inflammatory pathways in high-grade serous ovarian cancer
Source: Front Pharmacol. 2022 Aug 29;13:946348. doi: 10.3389/fphar.2022.946348 (PMC9465617; doi:10.3389/fphar.2022.946348)
Supplement: Supplementary file 1 [file DataSheet1.docx]

Supplementary Material

# Supplementary Tables

**Supplementary Table 1.** Summary of *SLCO4A1*/OATP4A1 mRNA and protein expression

| **patient** | **Original Materia**l*_1_ | **cell line** | **mRNA expression by RNA sequencing [read counts]** | **relative mRNA expression [RT-qPCR]** | **Western blot**  **(chemiluminescent intensity/volume of the band)*_2_** |
| --- | --- | --- | --- | --- | --- |
| 1 | T | 8587 | 2 | 0 | 397996 |
| 2 | A | 12370 | 287 | 115 | 73008 |
| 3 | A | 13363 | 5 | 0 | 0 |
|  | A | 15233_nov | 20 | 112 | 50485 |
| 4 | A | 13699 | 353 | 251 | 209475 |
| 5 | A | 13781 | 68 | 50 | nA |
| 6 | A | 13914 | 1359 | 656 | 216801 |
| 7 | A | 14433 | 2502 | 3167 | 860154 |
| 8 | A | 8540 | 1510 | 1499 | 0 |
| 9 | A | 15876 | 89 | 29 | nA |
|  | A | 16106 | 115 | 11 | 21090 |
|  | A | 17201 | 534 | 128 | 0 |
|  | A | 17268 | 21 | 10 | nA |
|  | A | EB | 34 | 10 | nA |
| 10 | T | 8684 | 668 | 412 | 213408 |
| 11 | A | 16471 | 1597 | 2416 | 90720 |
| 12 | P | 17142 | 862 | 260 | 0 |
|  | A | 17249 | 412 | 498 | nA |
| 13 | A | 17330 | 0 | 0 | 192150 |
| 14 | A | 17399 | 30 | 14 | 278110 |
| 15 | T | 8713 | 312 | 68 | 162877 |
| 16 | A | 17457 | 16 | 8 | 274113 |
|  | T | 8714 | 304 | 165 | 0 |
|  | P | EK_R1 | 72 | 29 | nA |
| 17 | A | 17480 | 1094 | 421 | 199797 |
|  | T | 8715 | 726 | 260 | nA |
|  | T | 8716 | 1070 | 344 | nA |
| 18 | T | 8724 | 20 | 5 | nA |
| 19 | T | 8732 | 352 | 107 | nA |
| 20 | A | 17978 | 466 | 750 | nA |
| 21 | A | 18483 | 73 | 25 | nA |
| 22 | A | 18507 | 1481 | 592 | nA |
| 23 | A | 18605 | 113 | 45 | nA |

*_1_: Original material: T: tumor tissue; A: ascites; P: pleural fluid.

*_2:_ nA: not available

**Supplementary Table 2. 77 selected genes**

|  |  | Mesothelial cells (N=5) | HGSOC (N=33) |
| --- | --- | --- | --- |
| ENSG number | Gene Name | Median | Median |
| ENSG00000109193 | *SULT1E1* | 24 | 6 |
| ENSG00000196502 | *SULT1A1* | 1192 | 240 |
| ENSG00000105398 | *SULT2A1* | 6 | 6 |
| ENSG00000137869 | *CYP19A1* | 6 | 2 |
| ENSG00000167165 | *UGT1A6* | 2 | 1 |
| ENSG00000171234 | *UGT2B7* | 0 | 212 |
| ENSG00000174607 | *UGT8* | 26 | 156 |
| ENSG00000242515 | *UGT1A10* | 0 | 0 |
| ENSG00000099377 | *HSD3B7* | 720 | 424 |
| ENSG00000203857 | *HSD3B1* | 806 | 0 |
| ENSG00000108786 | *HSD17B1* | 474 | 466 |
| ENSG00000130948 | *HSD17B3* | 1 | 4 |
| ENSG00000133835 | *HSD17B4* | 3847 | 2399 |
| ENSG00000025423 | *HSD17B6* | 80 | 35 |
| ENSG00000132196 | *HSD17B7* | 57 | 98 |
| ENSG00000204228 | *HSD17B8* | 142 | 213 |
| ENSG00000072506 | *HSD17B10* | 1366 | 1175 |
| ENSG00000198189 | *HSD17B11* | 763 | 646 |
| ENSG00000149084 | *HSD17B12* | 4258 | 4280 |
| ENSG00000170509 | *HSD17B13* | 1 | 6 |
| ENSG00000087076 | *HSD17B14* | 220 | 332 |
| ENSG00000099251 | *HSD17B7P2* | 44 | 64 |
| ENSG00000108785 | *HSD17B1P1* | 18 | 9 |
| ENSG00000101187 | *SLCO4A1* | 41 | 304 |
| ENSG00000091831 | *ESR1* | 69 | 716 |
| ENSG00000140009 | *ESR2* | 1 | 4 |
| ENSG00000101846 | *STS* | 1010 | 249 |
| ENSG00000118777 | *ABCG2* | 68 | 7 |
| ENSG00000095303 | *PTGS1* | 3054 | 5570 |
| ENSG00000073756 | *PTGS2* | 317 | 32 |
| ENSG00000125257 | *ABCC4* | 785 | 581 |
| ENSG00000134247 | *PTGFRN* | 6308 | 2446 |
| ENSG00000164120 | *HPGD* | 10 | 17 |
| ENSG00000143257 | *NR1I3* | 16 | 36 |
| ENSG00000174827 | *PDZK1* | 3 | 11 |
| ENSG00000141510 | *TP53* | 2802 | 2619 |
| ENSG00000148773 | *MKI67* | 3890 | 5796 |
| ENSG00000138798 | *EGF* | 65 | 34 |
| ENSG00000085563 | *ABCB1* | 44 | 2 |
| ENSG00000108846 | *ABCC3* | 403 | 215 |
| ENSG00000117528 | *ABCD3* | 1434 | 1873 |
| ENSG00000119688 | *ABCD4* | 1126 | 1059 |
| ENSG00000164163 | *ABCE1* | 3290 | 3306 |
| ENSG00000146648 | *EGFR* | 11235 | 3370 |
| ENSG00000105329 | *TGFB* | 2934 | 845 |
| ENSG00000106799 | *TGFBR1* | 2217 | 795 |
| ENSG00000163513 | *TGFBR2* | 3959 | 3011 |
| ENSG00000069702 | *TGFBR3* | 166 | 801 |
| ENSG00000112715 | *VEGFA* | 797 | 3517 |
| ENSG00000173511 | *VEGFB* | 1446 | 2164 |
| ENSG00000150630 | *VEGFC* | 3890 | 159 |
| ENSG00000017427 | *IGF1* | 103 | 68 |
| ENSG00000169083 | *AR* | 206 | 193 |
| ENSG00000174808 | *BTC* | 36 | 308 |
| ENSG00000196139 | *AKR1C3* | 66 | 130 |
| ENSG00000141736 | *ERBB2* | 2974 | 4738 |
| ENSG00000171791 | *BCL2* | 60 | 134 |
| ENSG00000183018 | *SPNS2* | 1424 | 701 |
| ENSG00000106392 | *C1GALT1* | 900 | 448 |
| ENSG00000086300 | *SNX10* | 488 | 76 |
| ENSG00000158555 | *GDPD5* | 534 | 626 |
| ENSG00000184261 | *KCNK12* | 7 | 3 |
| ENSG00000172159 | *FRMD3* | 43 | 55 |
| ENSG00000175782 | *SLC35E3* | 372 | 386 |
| ENSG00000180787 | *ZFP3* | 238 | 275 |
| ENSG00000196482 | *ESRRG* | 16 | 50 |
| ENSG00000137203 | *TFAP2A* | 375 | 1144 |
| ENSG00000156162 | *DPY19L4* | 620 | 1019 |
| ENSG00000144648 | *ACKR2* | 240 | 232 |
| ENSG00000242110 | *AMACR* | 56 | 39 |
| ENSG00000161533 | *ACOX1* | 1355 | 1969 |
| ENSG00000113161 | *HMGCR* | 934 | 3016 |
| ENSG00000117305 | *HMGCL* | 1064 | 847 |
| ENSG00000146151 | *HMGCLL1* | 3 | 8 |
| ENSG00000112972 | *HMGCS1* | 1272 | 4638 |
| ENSG00000135929 | *CYP27A1* | 416 | 52 |
| ENSG00000138135 | *CH25H* | 8 | 14 |

# Supplementary Figure


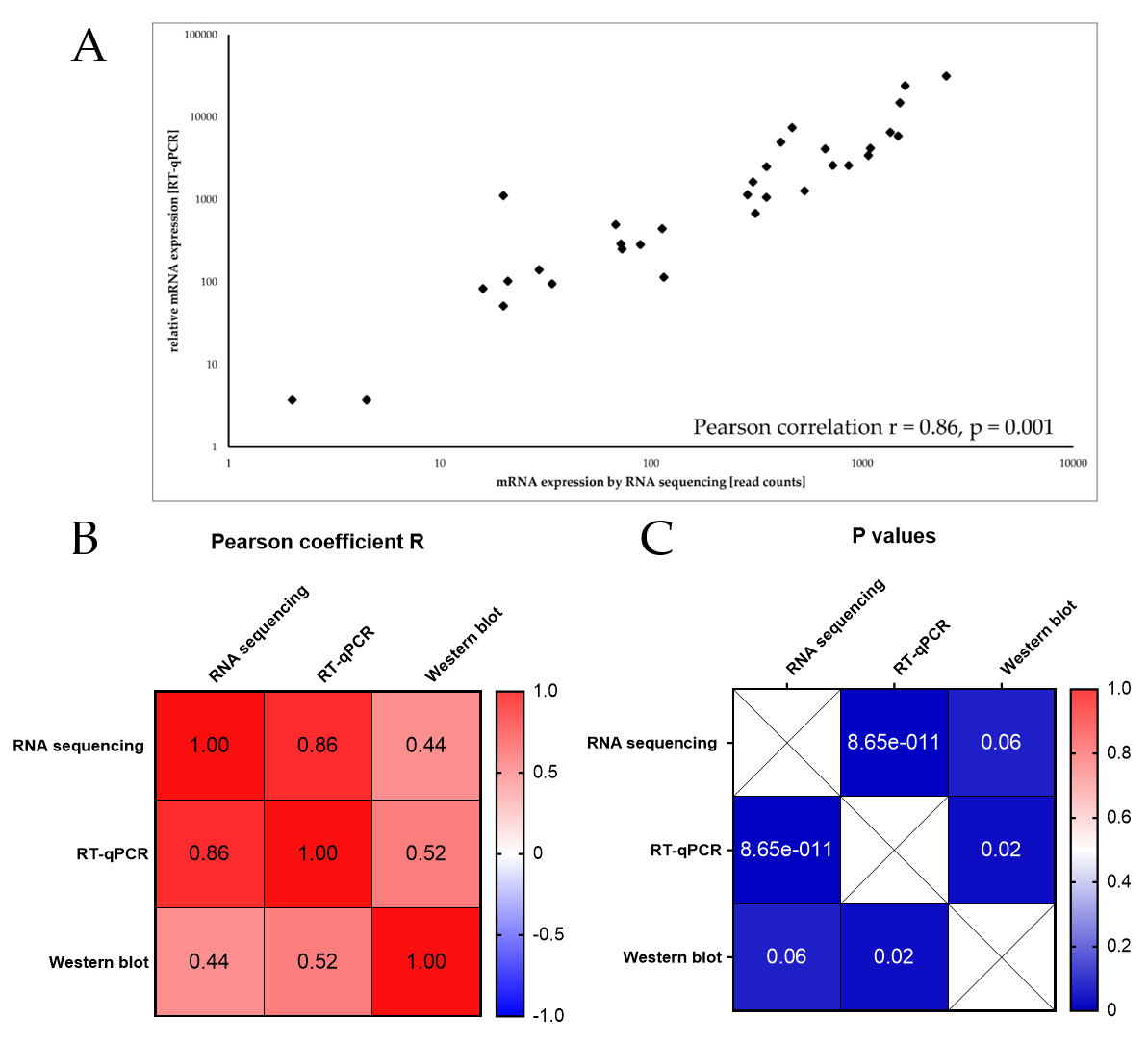


**Supplementary Figure 1.** Correlations of the mRNA expression determined by RNA sequencing, RT-qPCR and Western blot of *SLCO4A1* (A) Correlation of the mRNA read counts determined by RNA sequencing, and the relative mRNA expressions determined by RT-q PCR. (B) Correlation matrix, showing the Pearson coefficient. (C) The p-values of the correlation matrix in (B) are displayed.

.
